# Dietary polyphenol intake and risk of hypertension in the Polish arm of the HAPIEE study

**DOI:** 10.1007/s00394-017-1438-7

**Published:** 2017-05-04

**Authors:** Giuseppe Grosso, Urszula Stepaniak, Agnieszka Micek, Magdalena Kozela, Denes Stefler, Martin Bobak, Andrzej Pajak

**Affiliations:** 1 Integrated Cancer Registry of Catania-Messina-Siracusa-Enna, Via S. Sofia 85, 95123 Catania, Italy; 2 Department of Epidemiology and Population Studies, Jagiellonian University Medical College, Kraków, Poland; 3 Department of Epidemiology and Public Health, University College London, London, UK

**Keywords:** Hypertension, Dietary polyphenols, Flavonoids, Phenolic acids, Flavanols

## Abstract

**Purpose:**

Dietary polyphenols have been studied for their potential effects on metabolic disorders, but studies on risk of hypertension are scarce. This study aimed to test the association between total and individual classes of dietary polyphenols and incidence of hypertension in the Polish arm of the Health, Alcohol and Psychosocial factors In Eastern Europe (HAPIEE) study.

**Methods:**

A total of 2725 participants free of hypertension at baseline were tested for blood pressure or taking hypertensive medication within the last 2 weeks at 2–4-year follow-up visit. A 148-item food frequency questionnaire and the Phenol-Explorer database were used to estimate dietary polyphenol intake. Odds ratios (ORs) and 95% confidence intervals (CIs) of hypertension comparing the various categories of exposure (total and individual classes of polyphenol intake) with the lowest one (reference category) were calculated by performing age- and energy-adjusted and multivariate-adjusted logistic regression models.

**Results:**

During follow-up, 1735 incident cases of hypertension occurred. The highest quartile of total polyphenol intake was associated with 31% decreased risk of hypertension compared with the lowest intake (OR 0.69, 95% CI 0.48, 0.98) in women. There was no significant association in men. Among main classes of polyphenols, flavonoids and phenolic acids were independent contributors to this association. The analysis of individual subclasses of polyphenol revealed that, among phenolic acids, hydroxycynnamic acids were independently associated to lower odds of hypertension (OR 0.66, 95% CI 0.47, 0.93), while among flavonoids, most of the association was driven by flavanols (OR 0.56, 95% CI 0.36, 0.87).

**Conclusion:**

Certain classes of dietary polyphenols were associated with lower risk of hypertension, but potential differences between men and women should be further investigated.

**Electronic supplementary material:**

The online version of this article (doi:10.1007/s00394-017-1438-7) contains supplementary material, which is available to authorized users.

## Introduction

Polyphenols are a group of molecules contained in a wide variety of foods and beverages commonly consumed by humans [1]. These compounds are divided into five main classes according to their chemical structure: flavonoids, phenolic acids, stilbenes, lignans, and others [2]. Their absorption and bioactivity have been reported to vary with a great extent depending on their chemical structure [3]. Out of thousands identified phenolic compounds, only a limited number have been estimated to significantly contribute to daily dietary intake [4]. Nevertheless, despite high variability in inter- and intra-individual intake and absorption and great differences in bioavailability, polyphenols have been extensively studied in both epidemiological and experimental studies for their potential health effects due to anti-oxidant and anti-inflammatory properties [5 ].

A growing interest on association of dietary polyphenol intake with major cardiovascular risk factors has been the focus of recent research. Following in vitro and in vivo studies demonstrating significant effect in regulating inflammatory status [6], several epidemiological investigations tested the association between polyphenol intake and hypertension [7–14], although most of them only included dietary flavonoids [7–10] and lignans [11, 12]. Overall, studies reported inconsistent results. Findings may vary between studies because of differences in food composition tables and dietary assessment methodologies used. Given the documented heterogeneity in structural characteristics, bioavailability, absorption, and metabolism, it is of main interest to investigate the independent effect of each polyphenol group analyzed comprehensively. Moreover, foods and beverages contributing to their intake vary between countries in terms of both quantity and quality, which in turn may be responsible for most of the observed differences on consumption of individual polyphenol group. Current research on dietary polyphenol intake and CVD risk factors is promising, but not conclusive. Moreover, studies with comprehensive estimation of all major classes and subclasses of polyphenols are lacking and further evidence is needed. Thus, the aim of this study was to assess whether total, individual classes, and subclasses of dietary polyphenol intake were associated with average blood pressure and incident cases of hypertension in the Polish arm of the Health, Alcohol and Psychosocial factors In Eastern Europe (HAPIEE) study.

## Methods

### Study population

The HAPIEE study is a multicenter prospective cohort study investigating the role of biological, dietary, lifestyle, and environmental factors in cardiovascular and other chronic diseases in Eastern European countries [15]. The study protocol and the methodology used were reported in details elsewhere [15]. In the present study, only data from the Polish cohort were included. Briefly, a random sample of 10,729 individuals (aged 45–69 years) was recruited at the baseline survey (first wave) conducted in 2002–2005 (response ratio of 59%) in the urban area of Krakow, Poland. The participants provided written informed consent to complete a structured questionnaire and undergo a clinical examination. The present study was conducted on those individuals free of hypertension at baseline. Out of the 8822 individuals with available clinical information at baseline, a total of 2725 participants free of hypertension at baseline attended the last follow-up visit conducted in 2006–2008 and thus were included in the present study. Participants were followed for a median follow-up period of 4 years (range 3.2–5.4 years). Sample included in the analysis did not substantially differ in background characteristics or variables of interest concerning polyphenol consumption compared with the baseline examination (data not shown).

### Dietary assessment

Dietary data were collected using a 148-item food frequency questionnaire (FFQ) previously validated [16, 17]. Estimation of portion sizes was facilitated using photographic references. Participants were asked how often, on average, they had consumed that amount of the item during the last 3 months, with nine responses ranging from ‘never or less than once per month’ to ‘six or more times per day.’ Moreover, participants were asked to include additional drinks and foods and frequency of consumption by manual entry.

### Estimation of polyphenol intake

Data on the polyphenol content in foods were obtained from the Phenol-Explorer database (http://www.phenol-explorer.eu) [18]. The process of estimation of polyphenol intake has been described in details elsewhere [19]. Briefly, food items of the FFQ containing more food components were separated according to their ingredients and foods that contained no polyphenols were excluded from the analysis. The average food consumption was calculated (in g or ml) by following the standard portion sizes used in the study and then converted in 24-h intake. An advanced search was carried out in the Phenol-Explorer database to retrieve mean content values for all polyphenols contained in the foods obtained, and individual polyphenol intake from each food was calculated by multiplying the content of each polyphenol by the daily consumption of each food. Total polyphenol intake was calculated as the sum of all individual polyphenol intakes from all food sources encountered according to this process. In this study, we investigated exposure to total polyphenols and their main classes phenolic acids, flavonoids, stilbenes, and lignans; the main subclasses of phenolic acids, including hydroxybenzoic acids and hydroxycinnamic acids; the main subclasses of flavonoids, including flavanols, flavonols, flavanones, flavones, anthocyanins, and isoflavones; and “other” polyphenols, including alkylmethoxyphenols, alkylphenols, curcuminoids, furanocoumarins, hydroxybenzaldehydes, hydroxybenzoketones, hydroxycinnamaldehydes, hydroxycoumarins, hydroxyphenylpropenes, methoxyphenols, naphtoquinones, phenolic terpenes, and tyrosols.

### Demographic, lifestyle, and clinical measurements

Sociodemographic and lifestyle characteristics included age, gender, educational and occupational level, smoking, and alcohol drinking habits. Physical activity included energy expenditure in leisure time by reporting type and duration of activity according to the predetermined questionnaire items. The overall amount of energy expenditure was estimated in kcal/day and categorized in low, moderate, and high physical activity level. Individuals were categorized according their smoking status as (i) non-smokers and (ii) current smokers. Average alcohol consumption was categorized as (i) none or low (≤12 g/day) and (ii) alcohol drinker (>12 g/day).

Physical examination included measurement of height, weight, waist circumference, and blood pressure using standard procedures [15]. Body mass index (BMI) was calculated according to the formula weight (kg)/height (m)^2^. Blood pressure was measured three times at the end of the physical examination, and the final value was the mean among the second and third measurements.

### Ascertainment of hypertension and incident events

Participants were considered to have hypertension at baseline visit if they had a having systolic and/or a diastolic blood pressure measurement equal to or higher than 140 and 90 mmHg, respectively, or taking hypertensive medication within the last 2 weeks. The same procedures were applied at follow-up visits.

### Statistical analysis

Characteristics of the study cohort were described by baseline polyphenol consumption categories. Taking into account the natural differences in hypertension risk between men and women, gender-specific analyses were conducted. Descriptive presentation relied on cross tabulations. Continuous variables were presented as means and standard deviations (SDs), categorical variables as percentages. Variables were examined for normality (Kolmogorov test). Chi-square test was used for comparisons of categorical variables, Kruskal–Wallis test was used for continuous variables. The linear association between total and individual classes and subclasses of polyphenol intake and systolic and diastolic blood pressure measurements at follow-up visits was tested though linear regression analysis models. Odds ratios (ORs) and 95% confidence intervals (CIs) of hypertension comparing the various categories of exposure (total and individual classes of polyphenol intake) with the lowest one (reference category) were calculated by performing age- and energy-adjusted and multivariate-adjusted logistic regression models. Variables included in the multivariable model were age, total energy intake, body mass index, physical activity, educational status, smoking status, alcohol consumption, menopausal status (women only), and all main classes of polyphenols. When examining the association with total polyphenols, a sensitivity analysis was performed by including one at the time the major food sources of polyphenols based on our previous publication [20], to test whether the level of association was mainly driven by one individual food component. Statistical significance was accepted at *P* < 0.05. All statistical analyses were performed with SPSS for Windows 21.0 (SPSS Inc, Chicago, IL).

## Results

Baseline characteristics of individuals free of hypertension by quartiles of total polyphenol intake are presented in Table 1. There were no significant differences in the distribution of baseline characteristics by intake of total polyphenol with the exception of age (higher consumers were younger) and in total energy (in women only), sodium, and potassium intake (Table 1). Among total and individual classes of polyphenols, phenolic acids and their subclasses hydroxybenzoic and hydroxycinnamic acids were linearly inversely associated with systolic blood pressure measurements at follow-up visit in women but not in men (Supplementary Table 1). Also the group of other polyphenols was associated with lower both, systolic and diastolic blood pressure in women (Supplementary Table 1). In men, only stilbenes were linearly inversely associated with systolic blood pressure (Supplementary Table 1).

**Table 1:** Background characteristics of participants in the HAPIEE cohort free of hypertension at baseline by quartiles of total polyphenol intake

	Polyphenol quartiles men				*P* for trend	Polyphenol quartiles women				*P* for trend
	Q1	Q2	Q3	Q4		Q1	Q2	Q3	Q4	
No. of subjects	252	277	307	315		330	381	411	452	
Age (years), mean (SD)	57.4 (7.4)	56.2 (7.1)	56.1 (6.8)	55.4 (6.7)	0.008	55.7 (6.7)	55.8 (6.7)	55.0 (6.3)	54.4 (6.1)	0.005
BMI, mean (SD)	26.5 (3.2)	26.7 (3.4)	26.9 (3.6)	26.3 (3.4)	0.205	26.4 (4.3)	26.1 (4.2)	26.6 (4.3)	26.1 (3.9)	0.290
Baseline systolic blood pressure, mean (SD)	124.1 (9.7)	124.1 (9.1)	124.4 (8.8)	123.8 (8.9)	0.909	119.7 (10.5)	119.9 (10.9)	119.4 (10.7)	118.8 (10.7)	0.603
Baseline diastolic blood pressure, mean (SD)	77.8 (6.7)	78.6 (6.0)	78.4 (6.6)	78.3 (6.9)	0.592	76.7 (8.5)	76.8 (6.9)	77.0 (6.7)	76.7 (6.7)	0.922
Smoking status, *n* (%)					0.646					0.294
Smokers	108 (43.2)	118 (42.8)	125 (41.0)	145 (46.0)		106 (32.3)	134 (35.3)	127 (30.9)	164 (36.5)	
Non-smokers	142 (56.8)	158 (57.2)	180 (59.0)	170 (54.0)		222 (67.7)	246 (64.7)	284 (64.1)	285 (63.5)	
Educational level, *n* (%)					0.918					0.197
Low	20 (8.0)	21 (7.6)	23 (7.5)	20 (6.3)		31 (9.5)	43 (11.3)	33 (8.0)	33 (7.3)	
Medium	151 (60.1)	167 (60.3)	187 (60.9)	182 (57.8)		198 (60.4)	211 (55.5)	238 (57.9)	249 (55.1)	
High	80 (31.9)	89 (32.1)	97 (31.6)	113 (35.9)		99 (30.2)	126 (33.2)	140 (34.1)	170 (37.6)	
Physical activity level, *n* (%)					0.916					0.381
Low	65 (28.5)	74 (28.5)	77 (26.6)	78 (25.7)		97 (30.6)	115 (31.6)	105 (26.9)	117 (27.5)	
Medium	94 (41.2)	102 (39.2)	118 (40.8)	118 (38.8)		112 (35.3)	138 (37.9)	138 (35.4)	149 (35.0)	
High	69 (30.3)	84 (32.3)	94 (32.5)	108 (35.5)		108 (34.1)	111 (30.5)	147 (37.7)	160 (37.6)	
Alcohol consumption, *n* (%)					0.186					0.068
>12 g/day	244 (96.8)	265 (97.3)	289 (94.1)	293 (93.0)		325 (98.5)	371 (97.4)	393 (95.6)	443 (98.0)	
≤12 g/day	8 (3.2)	12 (2.7)	18 (5.9)	22 (7.0)		5 (1.5)	10 (2.6)	18 (4.4)	9 (2.0)	
Total energy intake (kcal/day), mean (SD)	1834.4 (529.6)	2049.4 (526.5)	2304.1 (632.8)	2557.5 (700.3)	0.205	1785.6 (524.7)	1969.2 (513.4)	2189.2 (576.1)	2359.4 (606.3)	<0.001
Sodium intake, mean (SD)	3096.4 (1069.2)	3354.6 (1125.8)	3685.3 (1261.5)	3829.9 (1280.3)	< 0.001	2859.6 (928.0)	3108.9 (931.9)	3302.2 (1072.2)	3563.2 (1140.5)	<0.001
Potassium intake, mean (SD)	3161.6 (989.4)	3494.8 (1025.4)	3897.9 (1332.9)	4343.9 (1372.4)	< 0.001	3025.3 (1017.8)	3415.3 (996.2)	3885.6 (1233.6)	4147.6 (1325.4)	<0.001
Fiber intake, mean (SD)	16.2 (5.3)	18.2 (6.1)	19.8 (7.9)	22.0 (7.8)	< 0.001	15.3 (5.5)	17.5 (5.7)	19.8 (7.0)	21.5 (8.7)	<0.001

During 4-year follow-up, 1735 incident cases of hypertension occurred. In the multivariate model, the highest quartile of total polyphenol intake was associated with 31% decreased risk of hypertension compared with the lowest (OR 0.69, 95% CI 0.48, 0.98; Table 2) in women but not in men. The sensitivity analysis by adjusting for major food sources of polyphenols did not change the retrieved associations (data not shown). Among main classes of polyphenols, flavonoids, phenolic acids, and other polyphenols were independent contributors to this association. The analysis of individual subclasses of polyphenol revealed that, among phenolic acids, hydroxycynnamic acids were independently associated with lower odds of develop hypertension (OR 0.66, 95% CI 0.47, 0.93), while among flavonoids, most of the association was driven by flavanols (OR 0.56, 95% CI 0.36, 0.87; Table 3). No significant associations were found in men.

**Table 2:** Odds ratios (ORs) and 95% confidence intervals (CIs) for the association between cumulative polyphenol intake (total and main groups) and incidence of hypertension

	Polyphenol quartiles, Men				Polyphenol quartiles, Women			
	Q1	Q2	Q3	Q4	Q1	Q2	Q3	Q4
Total polyphenols, mean (SD)	1024.9 (208.3)	1471.9 (103.1)	1866.9 (137.7)	2658.5 (586.1)	1035.8 (214.9)	1470.7 (101.3)	1878.5 (136.6)	2598.3 (559.9)
Incicent hypertension, *n* (%)	171 (67.9)	184 (66.4)	209 (68.1)	200 (63.5)	221 (67.0)	232 (60.9)	267 (65.0)	251 (55.5)
OR (95% CI)^a^	1	0.96 (0.66, 1.38)	1.07 (0.74, 1.55)	0.90 (0.61, 1.31)	1	0.77 (0.56, 1.05)	0.92 (0.67, 1.26)	0.62 (0.45, 0.84)
OR (95% CI)^b^	1	1.03 (0.66, 1.59)	1.07 (0.70, 1.65)	1.03 (0.65, 1.62)	1	0.80 (0.56, 1.14)	0.97 (0.68, 1.39)	0.68 (0.48, 0.87)
Flavonoids, mean (SD)	503.1 (121.1)	754.7 (55.3)	957.4 (69.5)	1474.1 (474.3)	504.0 (122.5)	758.1 (54.7)	956.9 (70.2)	1452.9 (482.0)
OR (95%CI)^a^	1	0.99 (0.98, 1.01)	0.94 (0.66, 1.35)	1.13 (0.76, 1.67)	1	0.99 (0.74, 1.34)	0.92 (0.68, 1.24)	0.77 (0.56, 1.06)
OR (95% CI)^b^	1	0.84 (0.53, 1.32)	0.93 (0.57, 1.51)	1.01 (0.57, 1.76)	1	0.91 (0.63, 1.31)	0.85 (0.58, 1.25)	0.64 (0.40, 1.01)
Phenolic acids, mean (SD)	284.2 (87.3)	614.4 (61.8)	841.2 (173.7)	1488.1 (301.4)	290.2 (86.3)	617.5 (60.7)	836.3 (171.5)	1450.9 (242.0)
OR (95% CI)^a^	1	0.94 (0.65, 1.34)	1.11 (0.78, 1.59)	0.88 (0.62, 1.25)	1	0.82 (0.60, 1.12)	0.80 (0.59, 1.10)	0.67 (0.49, 0.90)
OR (95% CI)^b^	1	0.96 (0.62, 1.49)	0.98 (0.64, 1.51)	0.92 (0.58, 1.44)	1	0.70 (0.49, 1.01)	0.75 (0.52, 1.08)	0.64 (0.44, 0.92)
Stilbenes, mean (SD)	0.005 (0.003)	0.017 (0.005)	0.046 (0.012)	0.525 (1.089)	0.004 (0.003)	0.017 (0.005)	0.046 (0.013)	0.575 (1.241)
OR (95% CI)^a^	1	0.99 (0.68, 1.45)	0.88 (0.61, 1.28)	0.88 (0.61, 1.25)	1	0.86 (0.64, 1.17)	0.81 (0.59, 1.09)	0.78 (0.58, 1.05)
OR (95% CI)^b^	1	0.98 (0.62, 1.56)	0.74 (0.46, 1.16)	0.71 (0.45, 1.12)	1	0.85 (0.59, 1.21)	0.77 (0.54, 1.09)	0.84 (0.59, 1.20)
Lignans, mean (SD)	0.16 (0.04)	0.24 (0.01)	0.31 (0.02)	2.09 (25.29)	0.16 (0.04)	0.24 (0.01)	0.31 (0.02)	1.86 (23.11)
OR (95% CI)^a^	1	0.84 (0.59, 1.20)	1.03 (0.72, 1.47)	1.03 (0.71, 1.47)	1	1.31 (0.98, 1.75)	0.94 (0.71, 1.25)	1.06 (0.78, 1.44)
OR (95% CI)^b^	1	1.06 (0.68, 1.65)	1.09 (0.68, 1.76)	1.29 (0.78, 2.16)	1	1.24 (0.88, 1.75)	1.05 (0.72, 1.52)	1.40 (0.92, 2.14)
Others, mean (SD)	6.1 (3.0)	17.0 (3.1)	32.9 (5.4)	77.3 (42.3)	6.56 (3.0)	16.7 (3.0)	32.6 (5.7)	76.8 (34.6)
OR (95% CI)^a^	1	1.01 (0.71, 1.43)	1.15 (0.81, 1.64)	084 (0.59, 1.18)	1	0.99 (0.73, 1.35)	0.75 (0.55, 1.01)	0.60 (0.44, 0.82)
OR (95% CI)^b^	1	1.18 (0.75, 1.87)	1.29 (0.81, 2.07)	0.87 (0.55, 1.36)	1	1.21 (0.83, 1.77)	0.87 (0.60, 1.26)	0.61 (0.42, 0.90)

^a^Age- and energy-adjusted

^b^Adjusted for age, total energy intake, body mass index, physical activity, educational status, smoking status, alcohol consumption (yes/no and continuous), sodium, potassium and fiber intake, menopausal status (women only), and all main classes of polyphenols included in the table

**Table 3:** Odds ratios (ORs) and 95% confidence intervals (CIs) for the association between individual polyphenol subclasses and incidence of hypertension

	Polyphenol quartiles men				Polyphenol quartiles women			
	Q1	Q2	Q3	Q4	Q1	Q2	Q3	Q4
Phenolic acids								
Hydroxybenzoic acids, mean (SD) (mg/day)	43.5 (24.2)	85.8 (0.9)	91.7 (3.6)	156.9 (834.2)	44.6 (22.9)	85.7 (0.9)	91.8 (3.9)	159.4 (35.8)
OR (95% CI)^a^	1	1.33 (0.87, 2.04)	1.02 (0.67, 1.55)	1.30 (0.85, 1.98)	1	1.07 (0.77, 1.48)	1.48 (1.05, 2.07)	1.04 (0.76, 1.45)
Hydroxycinnamic acids, mean (SD) (mg/day)	183.6 (73.9)	530.7 (65.6)	727.2 (177.3)	1391.2 (297.2)	196.7 (76.1)	534.2 (62.9)	716.3 (160.0)	1351.8 (229.2)
OR (95% CI)^a^	1	0.96 (0.62, 1.48)	1.11 (0.73, 1.69)	0.94 (0.62, 1.42)	1	0.85 (0.59, 1.21)	0.69 (0.48, 0.98)	0.66 (0.47, 0.93)
Flavonoids								
Flavanols, mean (SD) (mg/day)	324.1 (109.3)	520.2 (56.7)	681.8 (44.1)	1122.9 (427.6)	327.1 (106.5)	526.4 (58.6)	681.5 (44.2)	1089.1 (348.1)
OR (95% CI)^b^	1	0.74 (0.46, 1.17)	0.86 (0.54, 1.39)	0.81 (0.46, 1.41)	1	1.08 (0.74, 1.57)	0.68 (0.45, 1.01)	0.55 (0.35, 0.86)
Flavonols, mean (SD) (mg/day)	59.9 (15.0)	89.8 (6.5)	113.6 (7.7)	161.7 (34.5)	60.5 (14.8)	89.3 (6.2)	113.1 (7.7)	166.9 (65.7)
OR (95% CI)^b^	1	1.10 (0.69, 1.76)	1.49 (0.90, 2.45)	1.41 (0.79, 2.54)	1	1.05 (0.72, 1.54)	1.18 (0.78, 1.78)	1.43 (0.90, 2.27)
Flavanones, mean (SD) (mg/day)	24.9 (11.3)	59.9 (11.2)	103.4 (15.7)	221.4 (90.2)	25.3 (11.2)	60.1 (11.1)	106.7 (15.7)	216.5 (87.0)
OR (95% CI)^b^	1	0.91 (0.57, 1.44)	0.84 (0.52, 1.36)	0.88 (0.51, 1.54)	1	1.35 (0.96, 1.88)	1.52 (1.03, 2.24)	1.46 (0.96, 2.22)
Flavones, mean (SD) (mg/day)	1.9 (0.7)	4.3 (0.7)	7.7 (1.5)	16.4 (6.4)	1.9 (0.6)	4.2 (0.7)	7.9 (1.5)	16.0 (6.4)
OR (95% CI)^b^	1	1.02 (0.64, 1.62)	0.88 (0.53, 1.48)	0.92 (0.49, 1.73)	1	0.84 (0.59, 1.20)	0.84 (0.56, 1.26)	0.71 (0.4, 1.15)
Anthocyanins, mean (SD) (mg/day)	4.4 (2.7)	8.7 (1.2)	14.4 (2.4)	86.2 (133.5)	4.4 (1.9)	8.8 (1.2)	14.4 (2.4)	106.2 (234.0)
OR (95% CI)^b^	1	0.79 (0.48, 1.29)	0.86 (0.52, 1.41)	0.94 (0.55, 1.60)	1	1.52 (1.04, 2.21)	1.52 (1.03, 2.25)	1.31 (0.88, 1.94)
Isoflavones, mean (SD) (mg/day)	0.0005 (0.002)	0.140 (0.001)	0.196 (0.0002)	4.219 (6.287)	0.001 (0.001)	0.140 (0.001)	0.196 (0.001)	5.534 (11.172)
OR (95% CI)^b^	1	0.78 (0.46, 1.30)	1.01 (0.58, 1.77)	1.09 (0.63, 1.88)	1	1.12 (0.77, 1.64)	1.05 (0.68, 1.60)	0.91 (0.60, 1.38)
Dihydrochalcones, mean (SD) (mg/day)	1.8 (1.2)	6.8 (1.7)	9.7 (0.9)	25.1 (7.9)	1.9 (1.2)	7.0 (1.6)	9.7 (1.0)	24.9 (7.6)
OR (95% CI)^b^	1	0.94 (0.61, 1.46)	1.39 (0.85, 2.27)	1.19 (0.72, 1.96)	1	0.97 (0.68, 1.38)	1.01 (0.69, 1.47)	1.40 (0.93, 2.09)

^a^Multivariate analyses were adjusted for age, total energy intake, body mass index, physical activity, educational status, smoking status, alcohol consumption, sodium, potassium and fiber intake, menopausal status (women only), and phenolic acid subclasses listed in the table

^b^Multivariate analyses were adjusted for age, total energy intake, body mass index, physical activity, educational status, smoking status, alcohol consumption, sodium, potassium and fiber intake, menopausal status (women only), and flavonoids subclasses listed in the table

## Discussion

In this study, we investigated the association between total and individual classes of polyphenol intake and incidence of hypertension in urban population. We found that women with higher intake of polyphenols were less likely to develop hypertension compared to lower ones. Among specific classes and subclasses of polyphenols, higher consumption of flavonoids, phenolic acids, and “other” polyphenols was inversely associated with incident cases of hypertension. These results are in accordance with previous findings provided by a cross-sectional study conducted on the same cohort and testing the baseline relation between dietary polyphenol and presence of metabolic syndrome [21]; the study showed that among the main polyphenol classes, phenolic acids, flavonoids, and stilbenes were associated with lower odds of hypertension [21]. To date, only a limited number of epidemiological studies evaluated the association between total and individual classes of dietary polyphenols and cardiovascular outcomes, including hypertension [13, 14]. Despite no significant results were found in the analysis including total dietary polyphenol intake in a study conducted on Iranian adults, among individual classes sub-analyses revealed that phenolic acids and flavonoids were negatively associated with blood pressure, whereas flavonoids and stilbenes were negatively associated with hypertension [13]. Another study conducted in the context of the PREDIMED (PREvencion con DIeta MEDiterranea) cohort reported lower prevalence of hypertension among individuals with higher intake of dietary polyphenols [14] and further investigation in subgroup analysis revealed that also total polyphenols excretion was associated with blood pressure levels and prevalence of hypertension [22] as well as with plasma nitric oxide production, which is a well-known regulating factor of endothelial function [23]. Human diet contains a great variety of polyphenols but only some compounds and derivatives are bioactively relevant to endothelial function [24]. Moreover, differences in polyphenol food content databases and food items ascertained on dietary questionnaires may justify lack of significant findings in some of the aforementioned observational studies. In this study, we showed a wide picture of the possible association between polyphenol intake and hypertension, suggesting that independent associations of several polyphenol classes may exist, despite a concomitant and an overall synergic effect in preventing could be determinant in preventing hypertension. Up to date, this is one of the most comprehensive studies in terms of polyphenols investigated simultaneously considering hypertension as the outcome.

Results from this study may explain, at least in part, previous findings from meta-analyses of randomized controlled trials regarding the effect of flavonoids in lowering blood pressure, as they have been reported to be effective in hypertensive patients, but not in normotensive individuals [25, 26]. We reported that flavonoids were significantly associated with incident cases of hypertension when considered separately in the individual class analysis. Regarding subclasses of flavonoids, findings are contrasting and generally limited to some groups, such as anthocyanins [7] and isoflavone [8]. We did not found any significant result regarding these aforementioned subgroups of flavonoids, rather limited to flavanols. Literature on flavanol-rich food is wide, and findings from the few existing clinical trials show a potential blood pressure lowering effects of cocoa [27], while evidence on anthocyanins is contrasting [28, 29]. There is also evidence on the HAPIEE cohort that high adherence to dietary patterns rich in fruit and vegetable (among the main sources of flavonoids) were associated with better health outcomes compared to lower adherence [30–32]. Flavonoids have been hypothesized to exert beneficial effects in cardiovascular health by ameliorating the inflammatory status at various levels, for instance decreasing circulating levels of TNF-α and IL-6 [33]. Regarding their potential effects on blood pressure, flavonoids can act as inhibitors of endothelial NAD(P)H oxidase [34, 35], an enzyme implicated in the regulation of NO metabolism in the vascular endothelium, which in turn regulate vasodilatory processes associated with blood flow. Indeed, ingestion of pure epicatechin in humans has been demonstrated to increase NO bioavailability and to acutely reduce plasma concentrations of endothelin-1, which is a potent endothelium-derived vasoconstrictor [36]. Among other potential mechanisms of actions, flavonoids lower the activity of arginase-2, which is an enzyme that competes with NO synthase for l-arginine [37] and inhibit activity on angiotensin-converting enzyme in vitro [38].

Among the most studied hydroxycinnamic acids, chlorogenic acids (CGAs) (compounds richly contained in coffee) contained in coffee have been reported to exert beneficial effects towards CVD risk factors and metabolic disorders [39]. Meta-analyses of experimental studies CGAs reported significant reduced blood pressure compared placebo treatment [40], whereas results of observational studies on coffee consumption and hypertension showed a U-shaped association [41] and overall lower risk of metabolic syndrome [42]. Previous studies conducted on the HAPIEE cohort showed an inverse association between coffee consumption and metabolic syndrome and risk of hypertension [43, 44]. Together to their anti-oxidant properties, CGAs have been found to exert direct effects in regulation of blood pressure, as well as glucose and lipids metabolism [45]. CGAs have been hypothesized (i) to exert antihypertensive effects attenuating oxidative stress (reactive oxygen species) by reducing NAD(P)H-dependent super-oxide production and ameliorating endothelial dysfunction and (ii) to interact with the renin–angiotensin aldosterone system by inhibiting angiotensin-converting enzyme activity both in vitro and in vivo [46, 47].

Studies on stilbenes have been mostly focused on the effects of resveratrol on cardio-metabolic health [48, 49], revealing that resveratrol consumption significantly decreases systolic blood pressure at high dose [50]. We found inconclusive results on the risk analysis regarding the association between dietary stilbenes intake and hypertension risk, despite a linear association with individual intake and blood pressure measurements was found in men. Previous inconclusive prospective investigations on resveratrol efficacy on CVD outcomes suggested that the main limitation of studying stilbenes intake relied on the very small amount consumed [51]. The present study may be affected by similar limitation, as contribution of “normal” diets in stilbenes is generally negligible and, thus difficult to accurately estimate and associate with health outcomes [52].

Besides the most studied polyphenol classes, in this study, we opened the debate on other classes of polyphenols that may exert beneficial effects toward uncontrolled blood pressure and have not been previously explored in epidemiological studies. This study adds evidence that other polyphenols, such as tyrosol, may play an important role in decreasing the risk of hypertension. Studies on less studied polyphenols are highly warranted because they could explain the favorable effects of foods high in such compounds that have been inversely associated with hypertension risk. For instance, tyrosol and hydroxytyrosol contained in olive oil [53, 54] and some alcoholic beverages [55, 56] have been reported to reduce blood pressure, and may explain the beneficial effects related to cardiovascular prevention by consumption of the aforementioned food sources (moderate for alcohol). More research is needed on this topic, and further evidence will help to overcome potential limitation of observational studies on dietary polyphenol intake and health outcomes.

Strengths of the present study are prospective design, large size, and reliable assessment of individual diet. However, some methodological issues should be addressed when considering results from this study. First, we cannot conclude causation due to observational study design. Second, baseline evaluation of food intake may have introduced misclassification, because diet may have changed over the follow-up period. Third, certain polyphenol-rich foods, such as herbs and spices, may not have been entirely captured by the FFQ. Forth, use of table content databases would have inevitably led to some misclassification of polyphenol intake. However, such issues are common to all previous studies using the same methodology, and since polyphenol exposure was ascertained before diagnosis of disease, misclassification would tend to bias estimates toward the null and underestimate true associations. Finally, we found significant results only among women. A possible explanation is the natural differences occurring in men and women due to hormonal protection in the latter. However, potential differences need to be further investigated.

In conclusions, we reported that higher intake of some classes of polyphenols are associated with lower risk of hypertension. Results from the present study underline the importance of investigating all classes of polyphenols as possible determinants of health outcomes. Further studies are needed to clarify whether polyphenol classes consumed in small amounts may exert beneficial effects on health if consumed in higher concentrations and to establish the effects specifically attributed to each polyphenol class.

## Electronic supplementary material

Below is the link to the electronic supplementary material.

Supplementary material 1 (DOCX 33 KB)
