# Supplementary material for: Dietary polyphenol intake and risk of hypertension in the Polish arm of the HAPIEE study
Source: Eur J Nutr. 2017 May 4;57(4):1535–44. doi: 10.1007/s00394-017-1438-7 (PMC5959986; doi:10.1007/s00394-017-1438-7)
Supplement: Supplementary file 1 — Supplementary material 1 (DOCX 33 KB) [file 394_2017_1438_MOESM1_ESM.docx]

**Journal: European Journal of Nutrition**

**Dietary polyphenol intake and risk of hypertension in the Polish arm of the HAPIEE study.**

**Authors: Giuseppe Grosso^1,2^, Urszula Stepaniak^2^, Agnieszka Micek^2^, Magdalena Kozela^2^, Denes Stefler^3^, Martin Bobak^3^, Andrzej Pajak^2^**

^1^Integrated Cancer Registry of Catania-Messina-Siracusa-Enna, Catania, Italy, ^2^Department of Epidemiology and Population Studies, Jagiellonian University Medical College, Krakow, Poland, ^3^Department of Epidemiology and Public Health, University College London, London, United Kingdom.

**Corresponding author:**

Giuseppe Grosso MD, PhD, Integrated Cancer Registry of Catania-Messina-Siracusa-Enna, Via S. Sofia 85, 95123 Catania, Italy. Phone: +39 0953782182; Fax: +39 0953782177; Email: giuseppe.grosso@studium.unict.it

Supplementary Table 1. Association between individual polyphenol subclasses and systolic and diastolic blood pressure at follow-up.

|  | Men | | |  | Women | | |
| --- | --- | --- | --- | --- | --- | --- | --- |
|  | Beta | SE | P |  | Beta | SE | P |
| Total polyphenols |  |  |  |  |  |  |  |
| SBP | -0.15 | 0.66 | 0.814 |  | -1.01 | 0.57 | 0.080 |
| DBP | 0.43 | 0.37 | 0.238 |  | -0.02 | 0.31 | 0.938 |
| Flavonoids |  |  |  |  |  |  |  |
| SBP | -0.21 | 0.67 | 0.746 |  | -0.76 | 0.60 | 0.203 |
| DBP | 0.29 | 0.37 | 0.430 |  | -0.30 | 0.32 | 0.358 |
| Phenolic acids |  |  |  |  |  |  |  |
| SBP | 0.01 | 0.60 | 0.996 |  | -1.09 | 0.54 | 0.045 |
| DBP | 0.29 | 0.37 | 0.430 |  | 0.16 | 0.29 | 0.577 |
| Hydroxybenzoic acids |  |  |  |  |  |  |  |
| SBP | -0.28 | 0.60 | 0.644 |  | -1.24 | 0.54 | 0.023 |
| DBP | -0.04 | 0.34 | 0.905 |  | 0.49 | 0.29 | 0.100 |
| Hydroxycinnamic acids |  |  |  |  |  |  |  |
| SBP | 0.01 | 0.59 | 0.985 |  | -1.28 | 0.54 | 0.018 |
| DBP | 0.17 | 0.33 | 0.603 |  | 0.10 | 0.29 | 0.376 |
| Flavanols |  |  |  |  |  |  |  |
| SBP | -0.42 | 0.64 | 0.510 |  | -0.65 | 0.56 | 0.249 |
| DBP | 0.20 | 0.35 | 0.574 |  | -0.38 | 0.31 | 0.214 |
| Flavonols |  |  |  |  |  |  |  |
| SBP | -0.30 | 0.64 | 0.637 |  | 0.55 | 0.57 | 0.332 |
| DBP | 0.08 | 0.36 | 0.814 |  | 0.18 | 0.31 | 0.559 |
| Flavanones |  |  |  |  |  |  |  |
| SBP | -0.15 | 0.67 | 0.815 |  | 0.40 | 0.58 | 0.490 |
| DBP | 0.32 | 0.37 | 0.382 |  | 0.55 | 0.31 | 0.083 |
| Flavones |  |  |  |  |  |  |  |
| SBP | 0.78 | 0.76 | 0.304 |  | -0.62 | 0.65 | 0.342 |
| DBP | 0.80 | 0.42 | 0.058 |  | 0.10 | 0.35 | 0.762 |
| Anthocyanins |  |  |  |  |  |  |  |
| SBP | 0.20 | 0.65 | 0.754 |  | 0.04 | 0.57 | 0.934 |
| DBP | 0.20 | 0.36 | 0.577 |  | -0.12 | 0.31 | 0.693 |
| Stilbenes |  |  |  |  |  |  |  |
| SBP | -1.39 | 0.60 | 0.021 |  | 0.31 | 0.55 | 0.562 |
| DBP | -0.11 | 0.34 | 0.743 |  | -0.36 | 0.29 | 0.219 |
| Lignans |  |  |  |  |  |  |  |
| SBP | 0.05 | 0.60 | 0.923 |  | 0.31 | 0.55 | 0.562 |
| DBP | -0.24 | 0.34 | 0.473 |  | -0.07 | 0.30 | 0.808 |
| Other polyphenols |  |  |  |  |  |  |  |
| SBP | -0.12 | 0.59 | 0.832 |  | -2.23 | 0.56 | <0.001 |
| DBP | -0.34 | 0.33 | 0.301 |  | -0.90 | 0.30 | 0.004 |

SBP=systolic blood pressure; DBP= diastolic blood pressure
